# Ultrasonic neuromodulation as a new therapy for spasticity in an animal model of spastic cerebral palsy

**DOI:** 10.1590/acb394924

**Published:** 2024-08-16

**Authors:** Gisely de Andrade Costa Pereira, André Luiz Oliveira Poleto, Aldo José Fontes-Pereira, Marco Antônio von Krüger, Wagner Coelho de Albuquerque Pereira

**Affiliations:** 1 Universidade Federal do Rio de Janeiro – Biomedical Engineering Program – Rio de Janeiro (RJ) – Brazil.; 2 Universidade Federal do Delta do Parnaíba – Parnaíba (PI) – Brazil.; 3 Centro Universitário Serra dos Órgãos – Centro de Ciências da Saúde – Teresópolis (RJ) – Brazil.

**Keywords:** Muscle Spasticity, Child Development, Cerebral Palsy, Ultrasonic Therapy, Models, Animal

## Abstract

**Purpose:**

This study aimed to evaluate a new therapeutic option for the spasticity using ultrasound neuromodulation in an animal model of spastic cerebral palsy.

**Methods:**

Thirty-two adult male Wistar rats were randomly distributed in: negative control (NC); positive control (PC); untreated model (UTM); and treated model (TM). Rats in the control groups received sham surgery, and rats in the model groups received the spastic cerebral palsy model surgery. The rats’ motor functions were evaluated by the Rotarod and CatWalk tests before and after surgery. PC and TM groups underwent ultrasonic neuromodulation by a physiotherapeutic ultrasound (intensity 0.1 W/cm2, at 1 MHz) continuous mode for 5 seconds, for seven days.

**Results:**

Twelve rats showed a spastic pattern (UTM = 6 and TM = 6), motor limitations (UTM = 6 and TM = 6), and ten had difficulty feeding (UTM = 5 and TM = 5). One UTM group rat could not recover its preoperative latency time, while the other rats in the model groups did. The speed at which the limbs swung reduced after surgery and increased in subsequent assessments, demonstrating greater instability and a deficit in locomotion balance.

**Conclusions:**

Results were not yet sufficient to assert ultrasound neuromodulation as a possible therapy for spasticity in spastic cerebral palsy in the parameters used, and more studies are necessary.

## Introduction

According to the World Health Organization, nervous system disorders such as stroke, cerebral palsy (CP), Parkinson’s and Alzheimer’s are the leading causes of years of healthy life lost and the second leading cause of death worldwide, at around nine million deaths per year^1^. Among nervous system disorders, CP is the most common cause of lifelong physical disability, starting in childhood in most countries^2^. The prevalence of CP tends to be higher in low- and middle-income countries, reaching rates of three per 1,000 live births^3^^,^ 4.

CP is caused by an injury to the still immature brain, which occurs between the prenatal and postnatal periods (up to 2 years of age)^4^. It is a public health problem that transcends physical issues. The existence of a child with special health needs in the family can have financial repercussions^5^, due to the reduction or absence from work of a family member and the cost of treatment^5^^,^ 6, as well as socio-emotional repercussions, impacting on the quality of life of the whole family. In the United States of America, in 2003, the average cost of living was estimated at 921,000 dollars per person with CP^7^.

Among the types of CP, spastic cerebral palsy (SCP) is the most prevalent, affecting around 80% of cases^8^. This data is relevant, as spasticity is the motor disorder that most compromises the individual’s functionality, as it makes it difficult for them to position themselves comfortably, impairs tasks of daily living and limits the functionality of the affected limbs^8^^,^ 9.

The presence of spasticity also influences social participation and professional engagement, often leading to limitations in mobility and independence, contributing to loss of motor function, joint deformities, and chronic pain^8^^,^ 9. These restrictions can result in isolation and marginalization and negatively impact the individual’s physical, financial, and emotional health.

Despite the significant impact of spasticity on the lives of its sufferers, it is still a major challenge for medicine to treat it, mainly conservatively, i.e., without the need for surgery. Currently, treatment options involve surgical procedures, medication or approaches with physiotherapy, occupational therapy, or speech therapy. One of the bioethical ways of studying health treatments is to use animal models that simulate the condition in humans. Animal models help to understand physiology and pathological mechanisms, are predictive of results in humans and useful for assessing the safety and efficacy of treatments^10^.

Given the importance of studying spasticity, especially treatments to improve patients’ quality of life and health, this study aimed to analyze a new therapeutic intervention option using ultrasound neuromodulation based on clinical and motor data in an animal model of spastic cerebral palsy.

## Methods

### Experimental animal models

The experimental execution of this research took place after approval by the Animal Ethics Committee of the Universidade Federal do Rio de Janeiro under protocol no. 071-17, on August 30^th^, 2017. The research was carried out in accordance with the Guidelines for the Care and Use of Animals in Research and the national animal welfare legislation in force^11^.

### Sample

The study used 32 adult male *Rattus norvegicus* rats, Wistar strain, weighing 335 ± 29 g, with no previous contact with any experimental procedure, from the Central Animal Facility of the Health Sciences Center of the Universidade Federal do Rio de Janeiro (UFRJ). The animals were randomly distributed in four groups with eight rats each:

- Negative control (NC): rats without brain damage that did not receive therapeutic ultrasound neurostimulation;
- Positive control (PC): rats without brain damage that received therapeutic ultrasound neuromodulation;
- Untreated model (UTM): rats with brain damage that did not receive therapeutic ultrasound neuromodulation;
- Treated model (TM): rats with brain damage that received therapeutic ultrasound neuromodulation.

The animals were housed in the animal facility of the Neuroscience and Brain Improvement Laboratory at UFRJ, with free availability of food, controlled temperature conditions (22° C ± 2°C) and light (12-h light/dark cycle) in cages with up to four adult rats.

### Preparation of the spastic cerebral palsy animal model

**Figure 1:**
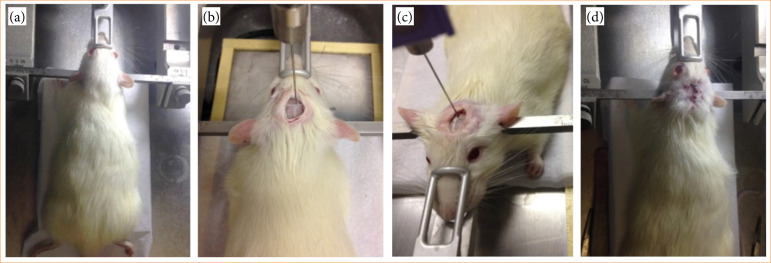
Procedure for preparing the animal model of spasticity. **(a)** Positioning the animal on the operating table and fixing it in the stereotactic apparatus. **(b)** Exposure of the brain sutures to position the needle and adjust the stereotactic coordinates. **(c)** Penetration of the needle into the cranial perforation for ethanol injection. **(d)** Animal after suturing the incision.

Source: Elaborated by the authors.

The experimental procedure for implementing the spasticity model (Fig. 1) followed Yu et al.’s^12^ protocol:

- The rats were anesthetized by intraperitoneal injection of 5–10-mg/kg xylazine associated with 60–90-mg/kg ketamine and 60–90-mg/kg alloxidine;
- The rats were positioned on the operating table and fixed in the stereotactic apparatus with a fixation bar in each ear for the surgical procedure;
- After anesthesia, the rats’ heads were trichotomized with a scalpel (no. 15), then disinfected with 70% alcohol and injected with the local anesthetic lidocaine;
- The SGE Analytical Science microsyringe, with capacity of 25 μL, was attached to the infusion pump support coupled to the stereotactic apparatus;
- A 2-cm-long parietal incision was made over the midline through the skin, subcutaneous tissue, deep fascia, and periosteum, layer by layer. After removing the periosteum, the area was cleaned with hydrogen peroxide, making it easier to identify the bregma and align the microsyringe needle;
- The meeting point between the frontal and parietal bone sutures and the sagittal suture was exposed. A 1-mm diameter hole was drilled in the skull, according to the positioning given by the stereotactic coordinates, 10 mm posterior to the bregma and 0.8 mm to the left of the sagittal suture, using a low-rotation dental drill. This hole was used to insert the microsyringe vertically to the depth of 9.7 mm;
- With the microsyringe filled with 15 μL of ethanol and positioned in the perforation according to the coordinates, the intracranial injection was made in the region of the pyramidal tract of the brain. In this study, a flow rate of 1 μL/min was set on the infusion pump;
- At the end of the injection, we waited approximately 2 minutes before slowly withdrawing the needle. The incision was cleaned and sutured;
- Healing ointment was applied to the incision, and the animals were injected with pentabiotics.

After the surgery, carried out at the Frontiers in Neurosciences Laboratory/Institute of Biomedical Sciences at UFRJ, the rats were assessed for changes in breathing, signs of pain or any adverse reaction. Immediately after the anesthetic effect (approximately after 2-hour postoperative), the animals were transferred and housed in a facility with a 12-h light/dark cycle. They had free access to water and food and were submitted to analgesic therapy (200 mg/kg of paracetamol dissolved in water, available in the drinking fountain) for one week.

All the rats underwent the procedures described. The rats in the NC and PC groups did not receive an ethanol injection after the needle had been inserted into the brain, following the stereotactic coordinates, but they did receive cleaning and suturing of the incision.

### Motor assessment

**Figure 2:**
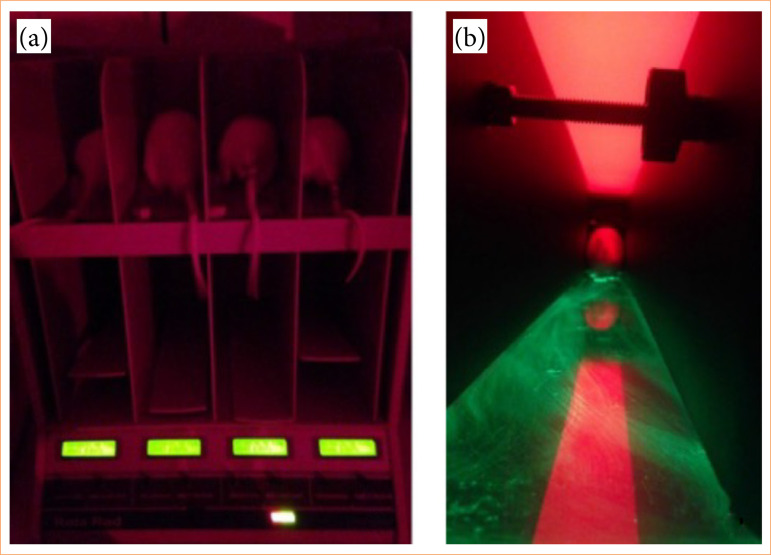
Rats during motor assessment. (a) Rats on the Rotarod. (b) Rat crossing the CatWalk walkway.

Source: Elaborated by the authors.

Coordination and balance tests with the Rotarod equipment and gait tests with the CatWalk method were used to assess motor function (Fig. 2). The assessments took place in the pre- and post-operative periods, after the rats had undergone five days of training before surgery. The motor assessment began 72 hours after the surgical intervention to induce brain damage, respecting the period of recovery and stabilization of symptoms^12^.

#### Rotarod

The Rotarod device (v. EFF 411, Insight Equipamentos, Ribeirão Preto, SP, Brazil) was used at the constant speed of 25 rpm for the maximum of 300 seconds (Fig. 2a). The latency time (or resistance time for the rat to fall or come out of the rod spontaneously) was recorded.

The Rotarod test was performed three times for each day, during 10 days of evaluation (one preoperative evaluation and nine evaluations after surgery). All tests were carried out at the same time of day with the lights off, only with the aid of a red light as described:

- The animals were transferred to the Rotarod room with the lights off, where they were acclimatized for at least 30 minutes before starting the test^13^;
- The speed of the rod was set at 25 rpm and remained constant throughout the tests;
- The animals were placed on the rod to walk, and the time spent on the rod was recorded after the animal fell or spontaneously left or when the time spent on the rod reached 300 seconds, which is the maximum time determined in the study^13^;
- After recording the time, the animal was transferred to the box and rested for 5 minutes to reduce stress and fatigue;
- The tests were carried out three times with each rat and the longest dwell time was selected for data analysis.

### CatWalk

The CatWalk software was used in this study to assess gait. CatWalk XT is an elevated walkway composed of a glass plate of at least 6-mm thick on which surface the rats walk on. The walkway is illuminated by an encapsulated fluorescent tube that is placed along the side edge of the glass so that the light enters the edge. This light is reflected inside the glass. Where the air is replaced by the rat’s paw (or other medium), the light leaves the glass plate due to the change in refractive index and is reflected, thus illuminating the paw.

A high-speed camera connected to a computer with CatWalk software (v. XT, Noldus Information Technology, Netherlands) is positioned under the glass walkway to capture the images of the rat’s steps that will be stored and later analyzed using CatWalk XT software.

The animals were trained in five sessions (one session per day for five days) before the first measurement (preoperative). To compose the analyzed data, three compatible runs were recorded on each evaluation day, with a minimum run duration of 0.5 seconds, a maximum duration of 60 seconds and a maximum speed variation of 30%. The camera was positioned 70 cm below the footbridge, and the distance between the side walls of the footbridge was set at 7 cm. The camera gain was set to 20 dB, the green intensity threshold was 0.10, the red light was 17.7 V, and the green light illuminating the walkway was 16 V.

The rats were assessed after training, once before and eight times after the surgical procedure, at a frequency of twice a week (Fig. 2b).

### Ultrasound neuromodulation

Ultrasound neuromodulation (UNMOD), using AVATAR III TUS0203 physiotherapeutic ultrasonic equipment (KLD Biosistemas Equipamentos Eletrônicos, Amparo, SP, Brazil), was carried out at the intensity of 0.1 W/cm^2^, continuous emission mode for 5 seconds, with a central frequency of 1 MHz, for seven days. The effective radiation area of the 1 MHz transducer is 3.08 cm^2^ and was obtained from its acoustic field^14^. It was applied transcranially to the area where the ethanol was injected.

To carry out the neuromodulation, a piece that reduces the contact area and improves contact with the rat’s head was attached to the transducer. Distilled water was placed inside the adapter to facilitate ultrasonic propagation, and water-soluble gel was applied to the head of the rat in which the transducer was positioned. The rats were immobilized with tissue, wrapping the entire body of the rat leaving only the head exposed to receive the ultrasonic irradiation.

UNMOD started 72 hours after the surgical intervention to induce brain damage, respecting the period of recovery and stabilization of symptoms^12^.

## Results

One animal in the UTM group died during surgery, which made it impossible to assess the success of the spasticity model protocol on the animal. As for the other animals, it was observed that 12 of the rats belonging to the model groups showed a spastic pattern (UTM = 6 and TM = 6), motor limitations (UTM = 6 and TM = 6), and 10 had difficulty feeding (UTM = 5 and TM = 5). A description of the rats’ clinical condition regarding their food intake, mental state, physical activity/mobility, the presence or absence of spasticity and the survival period after surgery is shown in Table 1.

**Table 1:** Clinical condition in terms of food intake, mental state, activity, spasticity, and survival after surgery to induce brain damage in rats by group.

Group	Rat	Food intake	Mental state	Activity/ Mobility	Spasticity	Postoperative survival
NC	1	Slight reduction until complete normalization in 72 hours	Slight depression until complete normalization in 72 hours	Practically normal after recovery from anesthesia in the postoperative period	No spasticity	-
	2					-
	3					-
	4					Died on the first postoperative day
	5					-
	6					-
	7					-
	8					-
PC	1	Slight reduction until complete normalization in 72 hours	Slight depression until complete normalization in 72 hours	Practically normal after recovery from anesthesia in the postoperative period	No spasticity	-
	2					-
	3					-
	4					-
	5					-
	6					-
	7					-
	8					-
UTM	1	Difficulty feeding independently	Depression of mental state with impact on mobility capacity	Rat without mobility, moving only limbs and lying down	With spasticity in right limbs	Died on the second postoperative day
	2	Slight reduction until complete normalization in 72 hours	Slight depression until complete normalization in 72 hours	Rat can walk and move, but with instability and misalignment	Spasticity in the left limbs, especially upper limb	-
	3	Slight reduction to complete normalization in 72 hours	Slight depression to complete normalization in 72 hours	Practically normal after recovery from anesthesia in the postoperative period	No spasticity	-
	4	Difficulty feeding independently	Depression of mental state with reflexes on mobility capacity	Rat became very weak, with little mobility, practically only moving its limbs without being able to assume a supportive posture, remaining lying down. He was only able to crawl for a short distance when he had some support (wall). Unable to stand on the Rotarod or cross the CatWalk	With spasticity in all four limbs	Died on the 14th postoperative day
	5	Difficulty feeding independently	Depression of mental state with impact on ability to move	Mouse without mobility, moving only the limbs and lying down	With spasticity in all four limbs	Died on the third postoperative day
UTM	6	Difficulty feeding independently	Depression of mental state reflected in ability to move	Rat without mobility, moving only its limbs, but with little range of movement. Only lying down	With spasticity in all four limbs	Died on the second postoperative day
	7	Difficulty feeding independently	Depressed mental state with impact on ability to move	Poor mobility, turning on its own vertical axis when trying to move	Spasticity in all four limbs, especially upper limbs	Died on the fourth postoperative day
	8	-	-	-	-	Died during surgery
TM	1	Unable to feed himself	Depression of mental state with impact on ability to move	Moved in a circle and very unstable in different postures	With spasticity in the right limbs	Died in the first postoperative day
	2	Slight reduction to complete normalization in 72 hours	Slight depression to complete normalization in 72 hours	Practically normal after recovery from anesthesia in the postoperative period	No spasticity	-
	3	Slight reduction until complete normalization in 72 hours	Slight depression until complete normalization in 72 hours	Rat can walk and move, but with instability and misalignment.	Mild spasticity in the lower limb	-
	4	Difficulty feeding independently	Depression of mental state with impact on ability to move	Rat with little mobility, remaining lying down	With spasticity in right limbs, mainly in upper limb	Died on the second postoperative day
	5	Difficulty feeding independently	Depressed mental state with impact on ability to move	Rat, when trying to move, rolled on its own vertical axis without being able to ambulate	With spasticity in all four limbs	Died on the third day
	6	Difficulty feeding independently, even after 72 hours of postoperative	Depressed mental state with impact on ability to move	Rat became very weak, with little mobility, practically only moving its limbs without being able to assume a supportive posture, remaining lying down. He only manages to crawl a short distance when he had some support (a wall). Unable to stand on the Rotarod or cross the CatWalk	Spasticity in the right limbs, especially in the lower limb	Died on the tenth postoperative day
	7	Difficulty eating independently	Depressed mental state with an impact on mobility	Poor mobility, lying down and basically moving his limbs	Spasticity in the right limbs, more pronounced in the upper limb. Presence of spasms in the left upper limb.	Died on the third postoperative day
	8	Slight reduction until complete normalization in 72 hours	Slight depression until complete normalization in 72 hours	Practically normal after recovery from anesthesia in the postoperative period	No spasticity	-

-: they survived until the end of the study; NC: negative control; PC: positive control; UTM: untreated model; TM: treated model. Source: Elaborated by the authors.

The animals’ latency time on the Rotarod was assessed on the same days as the CatWalk assessments. The rats’ latency times (in seconds) on the Rotarod in the preoperative and postoperative phases are described in Table 2.

**Table 2:** Latency time (in seconds) on the Rotarod in the preoperative and postoperative phases.

Group	Animal	Preoperative	Postoperative								
			1	2	3	4	5	6	7	8	9
NC	1	45	32	43	29	26	36	22	27	24	11
	2	300	300	300	300	300	271	300	300	208	300
	3	300	264	300	300	300	300	300	300	300	257
	5	300	300	300	300	300	300	300	300	300	300
	6	300	300	300	200	253	300	253	103	300	102
	7	300	112	243	240	300	300	300	300	300	300
	8	300	300	300	300	300	300	300	300	146	265
PC	1	300	300	300	300	300	300	300	300	300	300
	2	300	300	300	300	300	300	300	300	300	300
	3	300	300	300	300	300	252	252	224	300	300
	4	194	60	158	187	102	300	300	229	194	224
	5	300	300	300	178	270	300	300	300	113	300
	6	300	300	300	300	300	300	300	146	116	76
	7	237	300	177	268	270	300	300	300	300	300
	8	300	274	300	300	300	300	300	300	300	200
UTM	2	300	48	116	140	76	220	197	300	300	300
	3	300	189	291	235	50	54	72	67	21	56
	4	300	0	0	0	0	0	0	0	0	0
TM	2	249	223	215	300	300	300	300	300	300	249
	3	300	22	22	14	27	25	117	99	29	300
	6	300	0	0	0	0	0	0	0	0	0
	8	86	55	39	39	26	23	17	20	22	86

NC: negative control; PC: positive control; UTM: untreated model; TM: treated model. Source: Elaborated by the authors.

In the NC and PC control groups, 14 of the 15 rats reached a latency time of 300 seconds in the pre- and/or postoperative evaluations, with no major differences in time throughout the evaluations. Rat 6 in the PC group reduced its latency time in the last three evaluations. The longest latency time for rat 1 in the NC group, however, was 45 seconds in the preoperative assessment, and it maintained a shorter latency time in the other assessments. This rat spontaneously left the rotating rod of the Rotarod since the training period and maintained the same behavior.

In the UTM and TM model groups, one rat in each group (rat 4 UTM and rat 6 TM) was unable to perform the Rotarod test after surgery due to motor limitations, with a time of 0 second in the postoperative assessments. Rat 3 in the UTM group was unable to recover its preoperative latency time, while the other rats in the model groups did.

**Figure 3:**
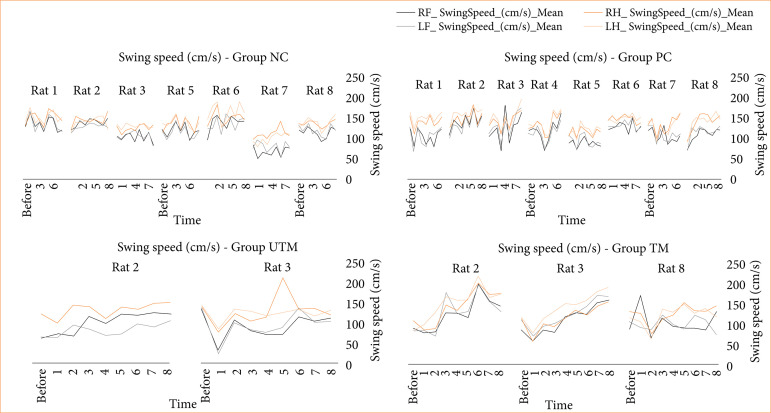
Swing speed (cm/s) of the limbs for all the paws of the rats in the NC, PC, UTM and TM groups.

NC: negative control; PC: positive control; UTM: untreated model; TM: treated model. Source: Elaborated by the authors.

**Figure 4:**
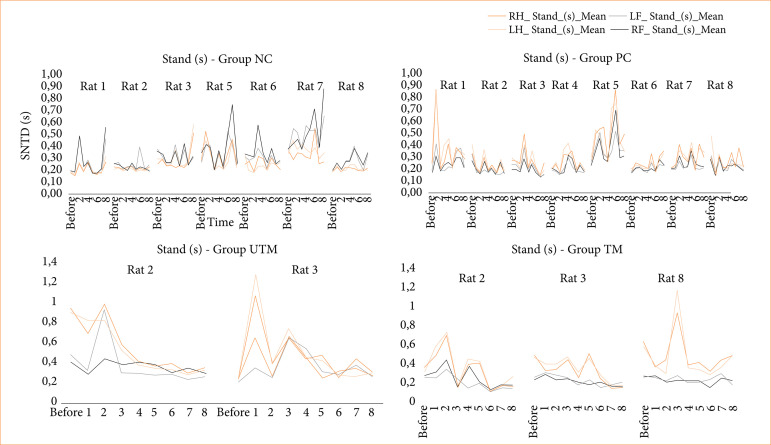
Duration of paw contact on the glass plate (stand) for all the paws of the rats in the NC, PC, UTM and TM groups.

NC: negative control; PC: positive control; UTM: untreated model; TM: treated model. Source: Elaborated by the authors.

**Figure 5:**
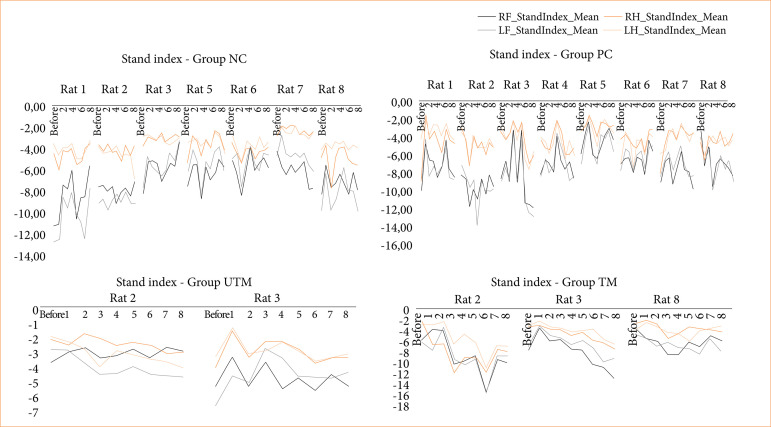
Speed at which the limbs lose contact with the glass plate (stand index) of all the paws of the rats in the NC, PC, UTM and TM groups.

NC: negative control; PC: positive control; UTM: untreated model; TM: treated model. Source: Elaborated by the authors.

**Figure 6:**
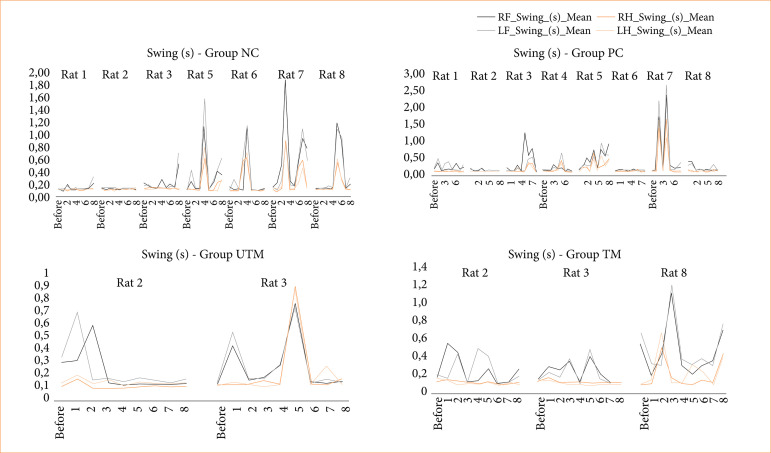
Swing time of the limbs of all the paws of the rats in the NC, PC, UTM and TM groups.

NC: negative control; PC: positive control; UTM: untreated model; TM: treated model. Source: Elaborated by the authors.

**Figure 7:**
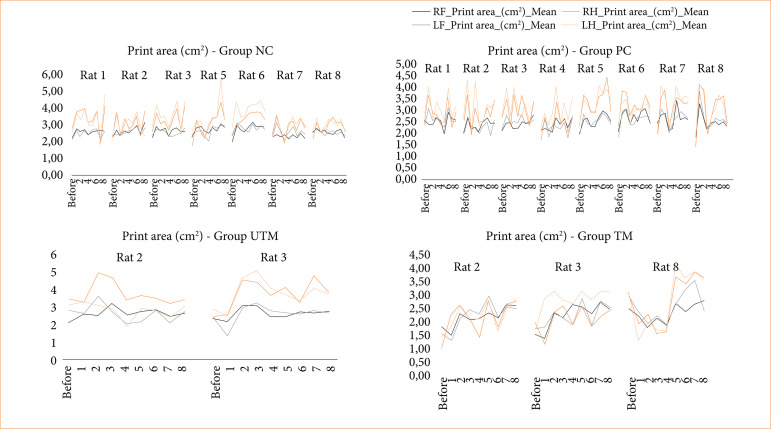
Footprint area of all paws of rats in the NC, PC, UTM and TM groups.

NC: negative control; PC: positive control; UTM: untreated model; TM: treated model. Source: Elaborated by the authors

**Figure 8:**
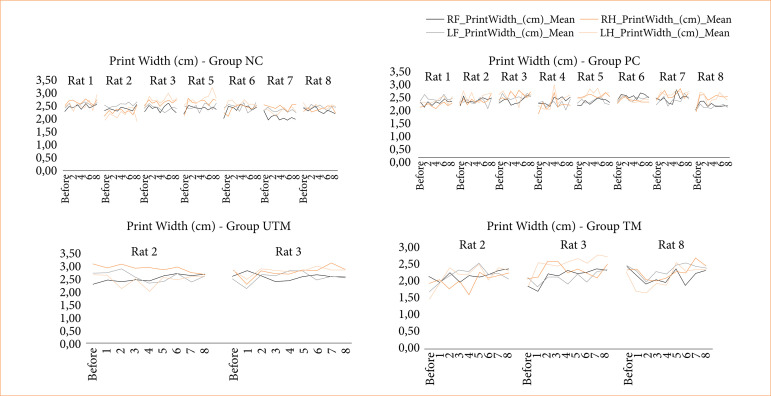
Paw print width of all paws of rats in the NC, PC, UTM and TM groups.

NC: negative control; PC: positive control; UTM: untreated model; TM: treated model. Source: Elaborated by the authors.

The data from the CatWalk test is shown in Figs. 3-8.

## Discussion

CP still has no cure and involves a series of health consequences, with varying presentation and severity. It can be classified into four types: spastic, ataxic, dyskinetic, and mixed. In any of the types, the main symptoms are related to motor impairments, which in this case are aggravated by spasticity. The impairments include changes in the gait pattern, ranging from increased energy and compensations to the inability to move independently. It is still a major challenge for medicine to treat spasticity, especially conservatively.

Treatments aimed at alleviating spasticity in SCP include surgical approaches, which are divided into neurosurgery or orthopedic surgery. The most commonly used neurosurgeries are dorsal rhizotomy, a procedure that involves selectively sectioning nerve roots in the spinal cord to reduce spasticity; intrathecal baclofen pump, which involves implanting a pump that releases the drug baclofen directly into the space around the spinal cord; and myelotomies and cordotomies, which are less-common procedures that involve interrupting certain pathways in the spinal cord. Orthopedic treatments generally involve procedures to improve the alignment of the bones and joints affected by spasticity.

Drug treatments include administration into the muscle itself, as in the case of botulinum toxin, or by other routes. The main substances used are baclofen, diazepam, tizanidine, clonidine and chlorpromazine, dantrolene and phenol. However, drug treatment does not achieve the desired result and is accompanied by side effects such as sedation, dry mouth, drowsiness, asthenia, dizziness, visual hallucinations, and hypotension.

Given this environment of traumatic treatments and/or treatments with insufficient or undesirable effects, this study intended to verify a new possibility for conservative treatment of spasticity looking for a positive impact on the functionality and consequent quality of life of people with SCP.

The proposal consisted of using therapeutic ultrasound as a neuromodulator of the spasticity present in SCP through its transcranial mechanical stimulation. To this end, we used the animal model of SCP proposed by Yu et al.^12^ with Wistar rats, which is easy to reproduce and provides long-lasting results of the spasticity typical of SCP.

The animal model used is advantageous compared to other models that simulate spastic cerebral palsy, as it offers the possibility of working with adult rats that are more tolerant of surgery, are affordable, easy to care for, as well as conducting behavioral tests. The method proposed by Yu et al.^12^ is simple to reproduce and capable of generating a chemical brain lesion in the area of interest, without causing direct damage to other areas. The authors targeted the lesion to the left pyramidal tract to generate spasticity and achieved obvious flexion spasms.

In previous experiments, the authors tested injecting other liquids to see if the cause of the lesion was the substance injected or the volume occupied in the rat’s brain. By using normal saline and hypertonic saline, they found that the rats showed no signs of flexion spasms. This fact suggested that the injury was not caused by the volume injected itself and that the level of brain damage depended on the substance injected^12^.

The authors stated that the symptoms remain for a long period and described the stabilization of symptoms within 72 hours. However, they did not specify how long the symptoms lasted and did not perform functional tests. They confirmed ethanol-induced brain damage in the region of interest (pyramidal tract) through histomorphometry performed 72 hours after induction of the lesion.

Histomorphometry is commonly used in biomedical studies, but its results can be affected by the quality of the data collected, including sample preparation, the quality of the imaging equipment and the experience of the researcher. The interpretation of histomorphometry results can be complex and requires a high level of knowledge and experience. In addition, histomorphometry can be a time-consuming process and requires significant resources, including specialized and costly equipment.

Yu et al.^12^ demonstrated the morphopathological validity of the model. In the present study, the same surgical technique was used to validate a reliable SCP model for the study of spasticity treatment possibilities, through clinical evaluation of the rats combined with the Rotarod and CatWalk tests, which provide information related to the rats’ motor skills. The aim was to verify the mimicking of the symptoms of SCP in the rats and the impact on their motor function, since improving function is the main purpose of the treatment of SCP with ultrasound neuromodulation.

The results of reproducing the animal model of SCP proved to be effective in mimicking spasticity, as reported by Yu et al.^12^ and Gyimesi et al.^15^. However, there was a significant loss of sample when considering only the model groups with brain damage (n = 16), in which 50% of the sample (n = 8) with ethanol-induced damage died between the first and fourth postoperative day. Yu et al.^12^ reported the loss of 16.6% of the sample (n = 2) during surgery due to anesthetic overdose, compared to 6.2% (n = 1) in the present study. Thus, the final number of animals in each group was NC = 7; PC = 8; UTM = 3; and TM = 4. Gyimesi et al.^15^ performed brain damage on four rats and did not report losses in the sample at any time.

The death of the animals may have been caused by the severity of the impairment produced by the brain injury associated with the animals’ difficulty in eating, which worsened their general condition. As the animals had difficulty with motor skills and feeding, some tablets of food were placed inside the box to make it easier for the animals to reach the food, and sometimes offered into the animals’ mouths.

The pattern of spasticity and motor impairment varied between the rats, and eight of them died within 96 hours of surgery or were so weak that they were unable to perform the motor analysis tests.

Future studies could test the same surgical technique, but with a reduced amount of ethanol to see if they could obtain a higher survival rate of the animals while maintaining the mimicry of the spasticity symptoms of SCP.

Among the 16 animals in the model groups with lesions, seven survived for more than four days, but only four of them had a clear presentation of spasticity, which they maintained until the end of the study. Of these four, two were unable to perform the Rotarod and CatWalk tests, as they were not mobile enough to stand on their paws and perform the tests. This fact does not exclude them as valid models for SCP because, just as in humans, SCP can have a varied topographical presentation and severity, including the inability to move independently.

The motor assessment used the Rotarod, which is a device designed to measure motor activity, indicating changes in balance and coordination. The Rotarod for rats consists of a rotating cylindrical rod 7 to 8 cm in diameter divided into compartments of equal size for simultaneous testing of several animals, a power source to rotate the roller and some kind of control of the speed of rotation of the rod.

The CatWalk method is currently widely used as an automated system for recording the running of rats and mice, making it possible to analyze various parameters of their gait and consequent motor function^16^.

In the Rotarod test, all the animals in the UTM and TM groups showed a drop in performance with reduction in latency time after brain injury, indicating changes in balance and motor coordination after surgery. The rats in the TM group that received UNMOD increased their latency time on the Rotarod over the course of the assessments, suggesting an improvement in the balance and coordination of the animals in this group.

In the UTM group which received no treatment, rat 2 improved its latency time on the Rotarod and rat 3 did not. Rat 2 did not develop the spastic pattern, and this probably favored recovery and improved motor performance after the brain injury. Rat 3, on the other hand, showed a spastic pattern and did not receive any therapeutic intervention and evolved with worsening motor performance in the Rotarod test.

Vandeputte et al.^17^ analyzed the gait parameters of rat models of Parkinson’s disease, Huntington’s disease, and stroke using the Catwalk method. They found statistically significant differences between the model and control groups in swing speed, duration of paw contact (stand), speed at which the limbs lose contact with the glass plate (stand index), swing time, grip intensity, print area, print width, and maximum area parameters.

In this study, no statistical tests were carried out to compare the control groups with the model groups, as the size of the groups changed after the surgical intervention to produce the model. However, when evaluating Figs. 3 to 8, changes can be seen mainly in the parameters of limb swing speed, duration of paw contact, speed at which the limbs lose contact with the glass plate and limb swing time throughout the evaluations, especially between the preoperative evaluation and the first post-operative evaluation.

The speed at which the limbs swung was reduced after surgery and increased in subsequent assessments, demonstrating greater instability and a deficit in locomotion balance after surgery. The speed at which the limbs lost contact with the glass plate was reduced, reinforcing the idea that the rats needed more contact time to stabilize and showed a deficit in motor function (strength, flexibility, balance).

Analysis of the impact of low-intensity therapeutic ultrasound neuromodulation was hampered by the size of the sample after brain injury. It is therefore recommended that further studies be carried out to establish an animal model for SCP, which, in addition to causing spasticity, has a higher survival rate. In this way, studies of possible therapeutic interventions can be carried out, and consistent results can be analyzed.

Once a reproducible animal model of SCP with the mentioned characteristics has been established, it is recommended that studies with a larger sample size be carried out with different UNMOD parameters and longer follow-up times to ascertain the therapeutic effect of UNMOD on the motor function of model rats.

The Rotarod and CatWalk tests proved to be sensitive to detecting motor changes before and after brain injury, with the Catwalk being more sensitive to more subtle changes, as stated by Vandeputte et al.^17^.

## Conclusion

Further studies are needed to establish a consistent animal model of SCP without significant animal loss for a period of more than 96 hours, which will enable the evolution of the models to be monitored and new therapeutic possibilities to be tested. With the limitations of the SCP animal model, the results of the study were not sufficient to safely indicate ultrasound neuromodulation as a possible therapeutic alternative for spasticity in SCP in the parameters used, although an improvement in clinical and motor findings was observed in part of the sample. More studies with therapeutic ultrasound are necessary to verify the UNMOD as an option to treat the spasticity in SCP.

## Data Availability

The data will be available upon request.
